# Personal and Social Consequences of Psychotropic Substance Use: A Population-Based Internet Survey

**DOI:** 10.3390/medicina58010065

**Published:** 2022-01-01

**Authors:** María Luisa Ballestar-Tarín, Vanessa Ibáñez-del-Valle, Omar Cauli, Rut Navarro-Martínez

**Affiliations:** 1 Department of Nursing, Faculty of Nursing and Podiatry, University of Valencia, Avda Menéndez Pidal 19, 46010 Valencia, Spain; M.luisa.ballestar@uv.es (M.L.B.-T.); Maria.V.Ibanez@uv.es (V.I.-d.-V.); Rut.Navarro@uv.es (R.N.-M.); 2 Frailty and Cognitive Impairment Organized Group (FROG), Department of Nursing, University of Valencia, Avda Menéndez Pidal 19, 46010 Valencia, Spain

**Keywords:** CRAFFT scale, alcohol, marijuana, consumption, substance, social consequences

## Abstract

*Background and objectives*: Drug abuse has become a major worldwide health concern among all age groups. The present study analyses substance misuse and its social and personal consequences using a population-based internet survey in Spain. *Materials and Methods*: Screening for drug abuse (of alcohol, marijuana/hashish and psychostimulants) and its related risks and problems was performed using the Car, Relax, Alone, Forget, Friends, Trouble (CRAFFT) score. Socio-demographic factors, depressive, anxiety and stress symptoms as well as health habits were also evaluated. We used Linear regression methods to compare each variable’s individual contribution so as to determine which one best explains the results. *Results*: In this population-based study, 1224 people completed and returned the online survey. Of all participants, 57% reported consuming at least one substance based on the CRAFFT scale. While increasing age reduces the probability of personal and social consequences of consumption, people who smoke receive up to three times more (OR = 3.370) recommendations from family and friends to reduce their consumption. As for the type of substance, the consumption of marijuana increases the risk of forgetting (OR = 2.33) and the consumption of other psychostimulant substances almost triples the risk of consuming alone (OR = 2.965). Combining substances can increase the rate of driving a vehicle after consumption by 3.4 times. *Conclusions*: Although age, smoking and the type of substances used increase the risk of suffering from social and personal consequences of the use or abuse of substances, future studies are needed to determine the influence of new variables as a potential tool for treating and minimizing the adverse consequences of drug abuse.

## 1. Introduction

Several studies show that experimentation with the effects of tobacco, alcohol and, to a lesser extent, other illegal substances begins during adolescence. Indeed, in recent decades, health agencies and university authorities have expressed concern about an increase in the use of alcohol and other substance abuse, e.g., of cannabis and amphetamines, among college students [1,2,3,4,5,6,7,8,9]. The abuse of these substances has become a major health concern worldwide, and although some adolescents who use or abuse these substances tend to reduce their consumption over time [10], it is plausible that a proportion of the adult population continues to use and abuse these substances. Several studies indicate that there is currently a relatively high risk of adults and/or older people maintaining problems with alcohol use, which can be combined with the use of other types of drugs [11,12,13,14], increasing the psychological, physical, economic [15] and social consequences [16]. Around 27% of Europeans aged 55 years and older report consuming alcohol on a daily basis. In addition, young people today drink more alcohol than previous generations, and it is to be anticipated that they will continue to do so as they age, leading to a higher prevalence of abuse of these substances in our elders of the future. Although the prevalence of illicit substances is higher among young people, there is an increase in the number of older adults who use them. Most older users of illicit substances are survivors because they begin this practice at an early age, and as with alcohol, are expected to continue their use into old age. In the United States, it has been estimated that up to 300% more older adults may use them by 2020 as a result of improvements in treatment and a declining overdose-related mortality rate [17,18]. In addition, the greater availability of these substances today is expected to accelerate their increased use among older users. At the same time, the use of substances such as marijuana is now so normalized that together with their relaxing and therapeutic effects, the growth in their use is understandable. There are multiple factors that contribute to the excessive consumption of alcohol [19,20,21], cannabis/marijuana, cocaine and tobacco, but in particular, they include the risk of presenting substance abuse behaviors due to changes in lifestyle [22], reduced socio-familial support [23,24,25] and the presence of stressful situations [26]. The consequences of alcohol misuse include physical health problems and poor academic performance [27,28]. For example, the health consequences of the abuse of alcohol, cannabis products and psychostimulants include alterations in the cardio-circulatory system [29,30,31], leading to tachycardia, whereby individuals with hypertension or heart failure may suffer worsening symptomatology. There are data indicating that there could be an increase in the incidence of anxiety crises, depression and psychosis due to chronic consumption of these substances among the many effects described on people’s health [32,33]. However, there is relatively little research that analyzes the relationship between substance use and the personal and social consequences of that use.

For example, among the social consequences, there is increasing data showing the detrimental effect of cannabis use associated with driving, especially when combined with alcohol, which increases the risk of accidents [34,35,36] and intimate partner violence [37]. Personal problems due to substance abuse include family [38], relational [39] and psychological [40] problems, and the loss of friendships and even jobs [41]. Moreover, these effects are quite frequent and independent of the type of drug used [42,43]. The CRAFFT Screening Test is a short and widely used clinical assessment tool designed to screen for substance-related risks and problems. The acronym stands for the key words of the six items “Car, Relax, Alone, Forget, Friends, Trouble” related to the most common (personal and social) problems related the consumption of such substances [44]. Among them, they are common social problems associated to the abuse such the concerns of family and friends (question Friends) or getting into trouble with other persons (Trouble) due to drug abuse. The items Relax, Alone and Forget are referred to the consequences of drug abuse on behavior to maintain the intake despite the individuals observed the need to improve the psychological well-being (Relax), the need to experience the effects of the drugs when being alone (Alone) despite the intake of this drugs could alter personal ability to manage the own daily life issues (Forget). The consumption of drug abuse to relax or feel better (Relax) is based on the fact that auto-referred stress is a well-known risk factor in the development of addiction and in addiction relapse vulnerability [45,46]. Given an impaired ability to stop or control intake, many individuals abusing these drugs find themselves drinking alone (Alone) and consuming them while being alone represents an important issue for development of addiction [9,47,48,49]. In fact, many people experienced first the effects of substances of abuse with other individuals and then, with or without company. Drug abuse is a serious factor in memory loss (Forget) and a lack of concentration, which can alter several aspects of daily life. The Car question represents a safety risk screener [50]. Internet-based survey studies are a useful tool for collecting information on drug-use patterns [50,51,52,53], they are quick and cheap to prepare, and can reach a large number of people who use drugs directly, i.e., it is a means of analysis that can provide a detailed and realistic picture of drug use and its personal and social consequences [54,55,56,57]

In this study, we had both descriptive and analytical objectives. The first, descriptive, was to estimate drug abuse prevalence and its social and personal consequences based on CFRATT screening tool in the Spanish population, based on internet-based survey. The second, analytical objective was based on the hypothesis that there are some socio-demographic factor influencing the use of these substances and the social and personal consequences (CRAFFT) associated with them. The following objectives were of the study:

Examine the use of alcohol, tobacco, marijuana/hashish and other illicit drugs.

Explore the social and personal consequences of substance abuse and the moderating effect of sociodemographic variables.

Study whether there is a relationship between this use and psychological factors such as stress, anxiety and depression.

## 2. Materials and Methods

### 2.1. Study Design and Population

This cross-sectional survey was conducted between November 2020 and February 2021. Exclusion criteria included being under 18 years of age and refusal to participate. Calculating the proportion of an infinite population with a precision of ±3%, and for a confidence level of 95%, a sample size of 1099 subjects was estimated. We increased this figure to 1224 subjects (10%) in anticipation of possible losses because the nature of the study of “drug abuse” means there could be losses, even if it is conducted anonymously for all participants. The calculation was performed with the Epi Info statistical package version 7.2. For the calculation of the sample size, an infinite population was considered. A population is considered infinite when the total number of observation units is unknown or the population is greater than 10,000 [58,59]. According to data from the National Institute of Statistics, in 2020 the Spanish population over 18 years of age was 39,180,773 [60].

### 2.2. Data Collection

Data were collected electronically using a self-administered questionnaire specifically designed for this study using Google Forms. The participants were recruited between November 2020 and February 2021. During this period, the questionnaire was launched via Facebook, Twitter and Instagram, which are the most globally popular social networks. For wider distribution of the survey, the survey link was subsequently sent via email through the researchers’ contact lists. Likewise, the survey was not scheduled to close when a sufficient number of people surveyed was obtained according to age or gender criteria. To know the time required to complete the online questionnaire, all members of the research team prior to its launch, filled out the survey and the time spent ranged between 8–12 min. The survey participants received no incentives.

The questionnaire gathered sociodemographic, lifestyle, and clinical data. The sociodemographic variables included age, gender, nationality, marital status and/or the existence of a stable partner, housing situation, and primary employment status. Regarding lifestyle, we asked about consumption and frequency of stimulant drinks (coffee, tea or cola), smoking, regular physical activity (30 min per day, 5 or more days per week) and sedentary periods of more than 2 h per day. In addition to indicating their weight (kg) and height (cm) for the calculation of BMI, the participants were asked to state whether they suffered from a chronic disease and if so, which one, as well as their self-perception of their overall state of health, using a scale of 1–10, where a higher score indicated a better self-perception of their health status. The participants also completed a package of validated self-assessment instruments on sleep quality, stress level, mood and substance abuse, as explained below.

### 2.3. Assessment of Substance Abuse

When assessing substance abuse (alcohol, cannabis and other illicit drugs, including illegal, over-the-counter and prescription drugs) and its consequences, we used the CRAFFT scale developed by Knight et al. [44] which has been validated in the Spanish language [61]. This scale is a brief, self-administered psychometric instrument designed to briefly and effectively identify individuals at risk for substance abuse. The CRAFFT questionnaire, with nine dichotomous response items (YES or NO), is divided into two parts. The first part, or part A, includes three questions on the use of alcohol, marijuana/hashish and other illicit drugs in the past 12 months. The second part, or part B, consists of six questions assessing problems related to the use of these substances. If the answers to the three questions in part A are “NO”, the respondent only answers the first question in part B of the questionnaire. However, if “YES” is answered to any of the three items in part A, the respondent must complete the entirety of part B of the questionnaire. The scores for each of these items ranged from 0 (NO) to 1 point (YES). The total score of the scale is obtained from adding the scores for the six items in part B. Scores equal to or higher than 2 points suggest abusive consumption, indicating the need for further assessment.

### 2.4. Self-Perceived Stress Level

Perceived stress was assessed using Cohen’s Perceived Stress Scale (PSS) [62] which has been validated in Spanish language [63]. The PSS is the best known instrument for measuring perceived psychological stress, i.e., the extent to which everyday life situations are appraised as stressful. This scale is composed of ten items used to assess how often the respondent feels a certain way during the last month. The responses are scored 0 (never), 1 (almost never), 2 (sometimes), 3 (quite often) or 4 (very often). However, items 4, 5, 7 and 8 are scored in reverse or inverted form. The total score ranges from 0 to 40 points, with higher scores indicating a higher degree of perceived stress.

### 2.5. Depressive and Anxiety Symptoms

We used the Goldberg Anxiety and Depression Scale to assess the level of anxiety or depression [64] which has been validated in Spanish language [65]. This is a questionnaire that not only orients the diagnosis towards anxiety or depression (or both in mixed cases), but also discriminates between them and quantifies their respective intensities. This scale consists of two subscales—one for anxiety and the other for depression. Each of the subscales is composed of nine dichotomous response items (YES or NO) to determine whether the respondent has had any of the symptoms mentioned in the last two weeks. The cut-off points are four or more points or affirmative answers for the anxiety scale, and two or more for the depression scale. The higher the score, the more severe the problem (the maximum possible score is 9 points for each of the subscales).

### 2.6. Ethical Considerations

This project was approved by the Human Research Ethics Committee of the University of Valencia (Valencia, Spain) (procedure number 1,232,539, dated 21 May 2020). Before participants could access the data collection instrument itself, they were provided with information about the study and had to explicitly express their wish to participate. Confidentiality and anonymity of the data were maintained through the online survey setup.

### 2.7. Statistical Analysis

A descriptive analysis of the sociodemographic variables was performed, showing the distribution of percentages for the qualitative variables, and the mean and standard deviation for quantitative variables. Non-compliance with normality was checked using the Kolmogorov-Smirnov test. The Mann-Whitney U and Kruskal-Wallis tests were used to compare means between the quantitative variables, and the Chi-square test for the comparison of proportions. Fisher’s exact test was used for comparisons in two-by-two tables. Spearman’s linear correlation coefficient was used to establish the correlation between the variables studied.

Logistic regressions were used to try to make a predictive model of consumption and the consequences of consumption. Logistic regression can be used to evaluate several factors simultaneously that are presumably related in some way (or not) to the dependent variable. Logistic regression analysis makes it possible to obtain measures of association (odds ratio) for each variable adjusted for the others and to detect possible interactions between them and the effect studied. First, logistic regression was performed to determine which sociodemographic and lifestyle variables are related to consumption, and how the variables that are associated with consumption are related to the consequences of consumption (CRAFFT-B items). Several regression analyses were carried out, the first to determine the variables that influence the consumption or not of substances. Subsequently, six logistic regression analyses were carried out, one for each of the items that constitute part B of the CRAFFT questionnaire, related to the consequences of consumption. These items (B1, B2, B3, B4, B5, B6) are dichotomous variables (0 = No and 1 = Yes). It is not done with the overall CRAFFT score. The interactions between independent variables are of concern, but the authors did not address this issue.

The level of statistical significance was established at *p* values below 0.05. The analysis was performed with SPSS 26 statistical software (IBM Corp, Armonk, NY, USA) licensed by the University of Valencia.

## 3. Results

### 3.1. Description of the Sample

The sample consists of 1224 participants who completed an online survey during the period from November 2020 to February 2021. The sociodemographic, lifestyle and substance use characteristics of the sample are presented in Table 1 and Table 2. A total of 192 men (15.7%) and 1031 women (84.3%) participated, with a mean age of 33.61 (±13.03). The marital status was: 51.9% single; 42% married/cohabiting with a partner; 5% divorced and 1.1% widowed. A total of 7.7% lived alone, 7.8% shared housing with people with no family ties; 17.8% lived with their partner and 66.7% lived with their family. The employment situation was as follows: 3.2% unemployed; 2.9% housewives; 35.2% students; 1.6% retired; and 57.1% active workers.

In relation to physical activity, 56.2% of the participants said that they performed regular physical activity (30 min a day, 5 or more days a week). However, 78.9% acknowledged having sedentary periods during the day lasting two or more hours. Furthermore, 64.3% of the participants were healthy adults, while 35.7% had been diagnosed with a chronic disease, while 55.3% had a normal BMI, 25.7% were overweight and 13.1% obese. The consumption of stimulant beverages was frequent in the population, with 76.9% of the participants saying they consume coffee (mean = 1.89 cups per day SD = 1.03), tea (mean = 1.33 cups, SD = 0.85) and/or colas (mean = 1.40, SD = 0.75). 16.9% of the participants smoke. Tobacco consumption was found to be similar among men and women (mean = 9 cigarettes; SD = 6.8) (*p* = 0.83).

As for consumption (CRAFFT-A), 57% of the sample reported consuming at least one substance, compared to 43% who said they did not. There are differences between men and women, with men consuming more than women (*p* < 0.006). These differences are present in the consumption of alcohol (*p* < 0.002) and other psychostimulant substances (*p* < 0.015).

### 3.2. Relationship of Substance Use with Sociodemographic and Lifestyle Variables

Substance use is associated with being male and with age. A total of 67.2% of men reported using harmful substances compared to 55.1% of women (*p* = 0.002). The older the participant, the less harmful substances consumed, while consumption is more frequent in participants of a younger average age (non-consumption 35.8 years, consumption 32.2 years) (*p* = 0.000). People who share housing with people without family ties (23.4%) consume more than those who live with their family (47.1%) (*p* < 0.000). Marital status influences consumption; consumption is higher among single people (60.4%) than married or cohabiting people (*p* < 0.042). The use of stimulant drinks (*p* = 0.002) and tobacco influences the consumption of psychostimulant substances (*p* = 0.000).

There is no relationship between consumption and physical activity (*p* = 0.552) or BMI (*p* = 0.953). However, consumption is higher among people who are sedentary, as 58.7% of sedentary people versus 50.6% of non-sedentary people consume (*p* = 0.023). It is also higher in healthy people (who do not have a chronic disease), at 59.5% versus 52.8% (*p* = 0.024).

As can be seen in Table 2, 57% of the participants report that they consume at least one harmful substance (alcohol, marijuana and/or another psychostimulant substance) The influence of the sociodemographic and lifestyle variables is different depending on the substance studied.

#### 3.2.1. Alcohol

Alcohol consumption is higher in men than in women, with 62.6% of men reporting consumption compared to 50.2% of women (*p* = 0.002). It is also higher in people who share housing with people without family ties (67%) than in people who live with their families (49.6%) (*p* = 0.009). It is related to people who drink stimulant beverages (*p* = 0.000) and who smoke (*p* = 0.000). There is no relationship between physical activity (*p* = 0.562) or a sedentary lifestyle (*p* = 0.068) in alcohol consumption. Consumption is lower among people with chronic disease (47.8%) than in healthy people (54.7%) (*p* = 0.023).

#### 3.2.2. Marijuana or Hashish

There are no differences in marijuana or hashish use between men and women (*p* = 0.37). Its use is more prevalent in single people (18.5%) than in married people (10.2%) (*p* = 0.000). It is not related to the consumption of stimulant drinks (*p* < 0.05), but it is related to tobacco use (*p* = 0.000). Overall, 30.2% of smokers consumed marijuana compared to 11.6% of non-smokers. There was no relationship with physical activity or a sedentary lifestyle. However, higher levels of consumption were observed in people with normal weight (18.4%) than in people with overweight (9.7%) or obesity (7.8%) (*p* = 0.000). Consumption is lower in people with chronic disease, at 11.8% vs. 16.3% (*p* = 0.034).

#### 3.2.3. Other Psychostimulant Substances

A total of 13.2% of men say they consume other psychostimulant substances, versus 7.6% of women (*p* = 0.015). Single people are more frequent consumers (*p* = 0.000). This consumption is related to smoking (15.1% of smokers vs. 7.1% of non-smokers) (*p* = 0.000). This consumption is not related to physical activity (*p* < 0.05), but it is related to sedentary lifestyles, as consumption is higher among sedentary people (9.4% vs. 5.1%) (*p* = 0.031). The percentage of consumers is higher in people with a chronic disease than in healthy people (11.8% vs. 6.7%).

### 3.3. Consequences of Substance Use

The descriptive statistics for the CRAFFT total score are shown in Table 2. The overall mean score is 2.97, with a standard deviation of 1.78. No gender differences are observed (*p* < 0.05). The mean score of the CRAFFT-B is 1.37 with a standard deviation of 1.48, with no significant differences according to sex.

As mentioned above, part B of the CRAFFT questionnaire refers to the personal and social consequences of substance use. The items with the highest percentage of responses are (in order) B5 (Have you ever FORGOTTEN what you did when taking alcohol, drugs or psychoactive substances?), to which 31.3% of the participants answered positively, B3 (Have you ever used alcoholic beverages, drugs or psychoactive substances to RELAX, to feel better about yourself or to integrate into a group?) with 29% of positive responses, B1 (Have you ever travelled in a CAR or vehicle driven by a person, including yourself, who has consumed alcohol, drugs or psychoactive substances?) answered positively by 24.5% of participants, and B6 (Have you ever consumed alcohol, drugs or psychoactive substances while ALONE and unaccompanied?) by 20%. The only significant differences between men and women were observed in items B2 and B4, where the percentage of positive responses was higher in men than in women, of 17.6% vs. 11.3% (*p* = 0.049) and 14.8% vs. 5.5%, respectively (*p* = 0.000).

Significant differences were observed according to age (Table 3). The consequences of consumption occur more frequently in the younger population; the largest age differences were observed for item B5 (27.45 years vs. 35.71 years). The social and personal consequences (CRAFFT part B) according to the substance used are represented in Table 4.

### 3.4. Mean CRAFFT B Score according to the Harmful Substance Used

The CRAFFT questionnaire takes into account the consumption of alcohol, marijuana and/or other psychostimulant substances. An analysis of their behavior showed that there were no differences between people who only consumed marijuana and those who consumed alcohol in addition to marijuana (*p* = 0.599), but there were differences with people who only consumed alcohol (*p* = 0.013). Likewise, the behavior is similar if other psychostimulant substances are consumed compared to those who combine them with other substances (alcohol and/or marijuana) (*p* > 0.05).

**Figure 1:**
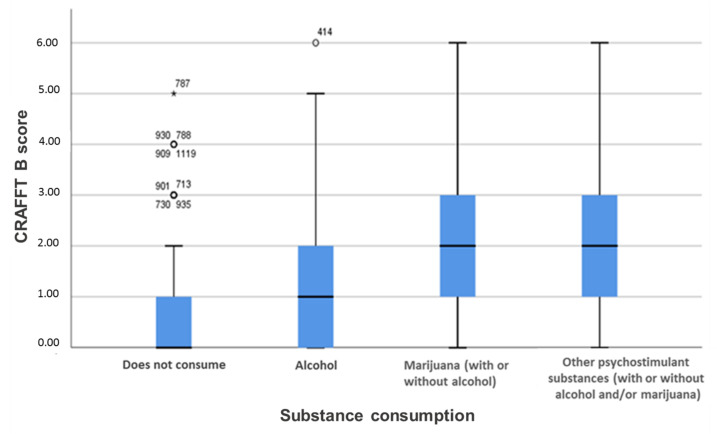
Substance use and consequences of its consumption.

Figure 1 shows the differences between the personal and social consequences of substance use. Significant differences are observed between non-consumption and consumption of any substance in the CRAFFT part B score. There are differences in scores between participants who drink alcohol and those who use marijuana with or without alcohol (*p* < 0.05) and between those who use alcohol and those who take other psychostimulant substances (*p* < 0.05). However, there is no difference in the CRAFFT-B score between those who use marijuana and those who use other psychostimulant substances (*p* > 0.05).

The consequences of substance use are evaluated using the CRAFFT part B score. The data are expressed as the mean ± standard deviation for each substance use.

Table 5 relates the consequences of consumption to the substance. The percentage of affirmative responses differs in items B1, B2, B3 between alcohol consumption and marijuana consumption (with or without alcohol) and alcohol and marijuana and/or other substances (*p* < 0.000). There are no differences between marijuana consumption (with or without alcohol) and the consumption of other psychostimulant substances (with or without alcohol and/or marijuana). In item B5, the percentage of affirmative responses was found to be higher in instances of marijuana use (with or without alcohol) (*p* < 0.000), while, in items B4 and B6 the percentage of affirmative responses is related to the use of other psychostimulant substances (with or without the presence of alcohol and/or marijuana) (*p* < 0.000).

### 3.5. Relationship between Substance Use, Perceived Health Status and Perceived Stress, Anxiety and Depression

The correlation between consumption (CRAFFT-A) and perceived health status is significant (*p* < 0.05), although the magnitude of the correlation is small (0.07). The correlation of consumption with stress, anxiety and depression is not statistically significant.

The mean score of self-perceived health among substance users differs statistically from those who do not use them (Table 6).

**Figure 2:**
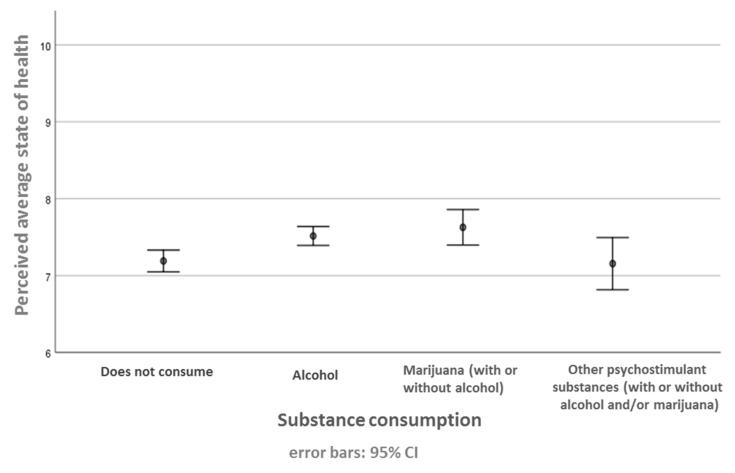
Perceived health status as a function of substance use. Perceived state of health was evaluated on a scale of 0–10. The data are expressed at mean ± standard deviation for each substance use.

There are differences between perceived health status and substance use (Figure 2) between the participants who report consuming alcohol and those who report not being consumers (*p* = 0.035). On a scale of 1 to 10, people who consume alcohol rate their health at 7.52 (SD = 1.34) compared to 7.19 (SD = 1.65) for people who say they do not consume alcohol. No differences were observed in the other categories (*p* > 0.05).

No relationship was observed between perceived stress and substance use (*p* > 0.05). There is also no difference in the score obtained in the Goldberg anxiety-depression questionnaire (global score) and substance consumption (*p* = 0.051). No relationship between the score on the depression scale and the substance consumed was observed.

However, when the influence of substance consumption was analysed with the anxiety subscale, differences were observed between people who consumed alcohol and people who consumed other psychostimulant substances (*p* = 0.032). The score on the anxiety subscale is higher among people who consume psychostimulants (in combination with other substances or not) than those who consume alcohol.

### 3.6. Variables Associated with Substance Use

A binary logistic regression model was applied to determine the influence of variables related to substance use. Based on the variables that were found to be significant in the previous analyses, the sociodemographic and lifestyle factors were analyzed to explain and predict consumption in the study sample.

To this end, a backward statistical procedure was applied, taking the multiple logit regression model as the initial model that includes the main effects of all the explanatory variables, and including consumption as a response variable with two categories: no substance consumption (category 0) versus consumption (CRAFFT A > 0 category 1).

Analysis of the initial regression model indicates that the variables Chronic Illness, Anxiety (Goldberg), Depression (Goldberg) and Stress (EEP-10) do not produce significant results (*p* = 0.989, *p* = 0.482, *p* = 0.631 and *p* = 0.867), and do not appear to be associated with substance use (CRAFFT-A).

The results of the variables included in the model are described below.

Sex has a statistically significant relationship with consumption. Being male increases the probability of consumption by 1.585. Increasing age reduces the risk, with the risk reduced by 0.025% for each year. Cohabitation is also related to consumption, with people who live in families consuming the least; the risk is multiplied by 2.336 when sharing a house with people who are not family members.

Substance use is also related to the consumption of stimulant beverages and tobacco, which increase the risk by 1.677 and 2.579, respectively. A sedentary lifestyle is also a risk factor, increasing the probability of consumption by almost 50%, although it did not reach statistical significance (OR = 1.499) (*p* = 0.058).

### 3.7. Variables Associated with the Consequences of Substance Use

The next step is to determine the variables associated with the consequences of substance use. Only people who consume at least one substance have been considered, since non-consumers did not answer part B of the CRAFFT questionnaire.

The variables included in the initial model are the same as those considered to determine whether or not the individual consumes substances (Table 7), with the addition of the variable of substance consumed (1 alcohol, 2 marijuana (with or without alcohol), 2 psychostimulant substances (with or without alcohol and/or marijuana) (Table 8).

The variables associated with each consequence vary (Table 9). Age influences behaviors B1, B4 and B5. In these cases, increasing age reduces the probability of the consequences. People who smoke in addition to consuming substances, receive up to three times more (OR = 3.370) recommendations from family and friends to reduce their consumption. The type of substance use is a risk factor in behaviors B1, B3, B5, B6. Combining substances can increase the risk of driving a vehicle after having consumed them by up to 3.4 times.

Marijuana use (with or without alcohol) increases the risk of forgetfulness (CRAFFT item B5) by 2.33 times and the use of other psychostimulant substances (with or without alcohol and/or marijuana) almost triples the risk of solo use (OR = 2.965).

## 4. Discussion

The main objective of Internet surveys investigating drug use behavior is to obtain valid and accurate measures of drug use in the population [66], and in our study, to obtain information on the personal and social consequences of drug use. Tools that use the Internet as an effective communication channel are an alternative for addressing the global problem of substance abuse, as this tends to be underreported [67]. A large number of studies conducted in recent decades have quite clearly underlined that self-administered questionnaires are more appropriate than other modes of data collection for collecting reports on sensitive behaviors such as this one [66,68]. Indeed, responses to self-administered questionnaires, whether completed with pen and paper or via a laptop computer, appear more reliable, and are particularly suitable for reporting behaviors that may compromise the respondent in some way, such as intimate or painful feelings, or illegal behaviors [69,70]. This could be attributed to the absence of a direct, identifiable witness, which therefore ensures the respondent’s anonymity and greater freedom of expression on personal topics such as this [71].

A large percentage (almost 80%) of Spanish families have an Internet connection, and 64.3% of the population uses the Internet on a daily basis [72] making it a crucial tool for population-based surveys as in the present study.

Our study showed that among the personal consequences of substance use, the item with the largest percentage of positive answers referred to forgetting personal experiences during use: “Do you ever FORGET things you did while using alcohol or drugs?”. This result is supported by the well-known effects of substance abuse on the neurophysiology of brain circuits related to memory, such as those involving the hippocampus [73,74,75,76]. Study in humans have shown that marijuana use alters multiple brain functions, including memory, as shown by the deficit in working and short-term memory related to its use [77]. Cannabis users showed poorer learning and memory than tobacco users, suggesting an effect of long-term cannabis use on memory despite short-term abstinence [78,79]. With respect to the effects of alcohol exposure on cognition, at higher doses it affects the performance of cognitive tasks that depend on the hippocampus, whereas at low doses it has no effect or even facilitates working memory when performing certain tasks [76]. Among the personal consequences, the item “Do you ever use alcohol or drugs to RELAX, feel better about yourself, or fit in? “ is the second most rated in our study. This use could be influenced by social culture, since some substances such as marijuana have been used worldwide for medical, recreational and spiritual purposes for thousands of years [80], and in the particular case of cannabis, because of the anxiolytic effects derived from its consumption [81]. A higher proportion of users of marijuana and/or other psychostimulant substances (with or without alcohol) reported “using to relax”. Although people tend to have multiple reasons for using substances, these results would be in line with those found in various studies on the motivational aspects of marijuana use, in which among the main reasons for use are to relax, wanting to feel better and to forget problems [82]. This is alarming given that Patrick et al. [82] found that motives related to Get High + Relax Reasons are considered potential reasons to continue consumption after the substance has been tried. Meanwhile, alcohol is a legally permitted substance that is widely consumed throughout the world, in both social and cultural settings [83]. This widespread use and its extensive use in social settings would justify the responses in this study that relate substance use to social integration. In a study published by Skobic et al. [84], the authors conducted a telephone interview with a sample size similar to this study of 1639 participants, and they found a high prevalence of marijuana use for sleep induction/relaxation. Substance use and its use to relax or “feel better” may also be related to the presence of associated comorbidity, such as major depressive disorder (MDD). This disorder can profoundly alter the patient’s social, family and work performance, and is the most prevalent comorbid mental disorder among people with substance use disorders [85]. Studies such as that of López and Becoña [86] evidence a correlation between cocaine use and depressive symptomatology. The results of our study found a trend towards a significant association between the score obtained in the Goldberg anxiety-depression questionnaire and substance consumption (*p* = 0.051). The results of our study for the consumption of psychostimulants for relaxation purposes may have been influenced by the existence of comorbidity related to anxiety, since the score on the anxiety subscale was higher in people who consume this type of substance. One third of the participants in our study say that they have at some point forgotten what they did when they drank alcohol, or took drugs or other psychoactive substances. This is the most frequently reported personal and social consequence of drug consumption, although it is strongly modulated by age [61].

Alcohol intoxication produces effects that can impair judgment and decision making, and increase participation in risky behaviors including drunk driving [87,88,89]. The study by Motschman et al. [87] reported that individuals who are under the influence of alcohol may perceive themselves as “unsafe” to drive, but “safe enough” to drive short distances, especially when their blood alcohol concentration is decreasing. Driving under the influence of alcohol or other substances is one of the most frequent consequences of alcohol use in our sample, with approximately 30% of the participants reporting having driven under the influence of at least one substance. In our study, this behavior is not related to gender, while other studies indicate that these risk behaviors are more frequent among males than females [90,91]. The frequency of this behavior decreases with age, although these risky driving behaviors are related to the perception of low risk and impulsiveness of youth [92]. The type of consumption influences the consequences of consumption; alcohol with another substance (marijuana or another psychostimulant) can therefore triple the risk. Sewell et al. [93] note that the combination of alcohol and cannabis results in greater impairment even at doses that would be negligible if they were of either drug alone.

Regarding the differences in personal and social consequences according to age, the results of our study show that forgetfulness is more frequent among young people. This could be due to a pattern of consumption with an increasing trend in this population group, as reflected in the 2021 Report issued by the Spanish Observatory on Drugs and Addictions [94]. According to this report, the prevalence of consumption (last year) is higher in the 15-34 age group, and in the case of alcohol, binge drinking has generally experienced an upward trend since 2007. Heavy drinking and binge drinking are more prevalent among males and are more heavily concentrated in the 15–34 age group. The alcoholic amnesia that causes forgetfulness occurs when high blood alcohol levels are reached in short periods of time, and are more related to the speed of intake than to the quantity [95].

As for the differences in personal and social consequences according to age, older people drink alone more often than younger people. In Spain, alcohol consumption in young people is most commonly associated with the “botellón” phenomenon. Among young people, there has been a shift away from a “Mediterranean” model of alcohol consumption, associated with meals, to an “Anglo-Saxon” model characterized by consuming drinks with higher alcohol content, in larger quantities and over shorter periods of time [96]. As for illicit psychostimulants, these are used more in specific subgroups or cultures according to Favrod-Coune and Broers [97]. Cannabis is the most widely used illicit drug among adolescents and young adults [98], and its effects have traditionally been considered less harmful than those associated with the abuse of alcohol and other illicit substances (e.g., cocaine and amphetamines) and injecting drugs [99]. According to data published by the Spanish Observatory on Drugs and Addictions [94], young people generally have a low perception of the risks associated with cannabis use, and in addition, after alcohol and tobacco, it is the psychoactive substance perceived as the most available by this population. These factors could influence the minimization of the adverse effects derived from the use of some substances among young people and their widespread use in social settings. Furthermore, in our study, living together as a family constitutes a protective factor for consumption, so that young people could limit their use of toxic substances to leisure time in the company of other young people. Confirming the results about the effect of age on abuse pattern, increasing age represents a protective factor. This may be due to the social roles associated with increased opportunities for exploration and experimentation among the younger population [100], as opposed to the social commitments of adulthood, such as being married, having children, and working full-time [101]. Although the family environment can act as a risk factor in the consumption of harmful substances, in our study, people who live in families, as opposed to those who share housing with people with whom they have no family ties (or friends), presented a lower risk of consumption. This could be in line with Brook et al. [102], who state that there are protective factors in the family environment such as conventionality, balance, maternal adjustment and strong attachment bonds in the family, which can mitigate the vulnerability of children to drug use when they relate to friends and peers who consume drugs.

We observed that being male increases the probability of consumption for almost all substances (except for marijuana/hashish, which was similar for both males and females). This finding, together with those systematically reported in previous studies [103,104], shows how the use of harmful substances continues to be an issue related to masculinity. This could be linked to the social mandates that men receive from birth, such as risk-taking or avoiding showing their emotions as ways of publicly affirming their masculinity [105]. Another possible explanation for this finding is the greater social rejection of illicit drug use among women [8]. In addition, the higher prevalence of impulsivity traits observed among males [106] and the fact that impulsivity is associated with a predisposition to the use and abuse of toxic substances and the pursuit of risky behaviors [107] could also partially explain a higher consumption of harmful substances among the male population. However, it is important to note that in recent years, a trend towards a homogenization of consumption patterns among both sexes, particularly among the younger population, has been observed in most studies [8,108]. In addition, we found that certain health habits such as smoking tobacco and the intake of stimulant beverages increase the likelihood of substance use. Previous research has generally shown that tobacco use alone predicts both alcohol abuse and illicit drug use [109,110]. These results could be explained by the gateway hypothesis, which holds that the use of a legal and easily accessible harmful substance, such as tobacco, increases the risk of starting to use another, probably more harmful, drug [111]. Meanwhile, a systematic review conducted by McKetin et al. [112] revealed that combining alcohol with caffeinated energy drinks increased the desire to continue consuming more alcohol. Approximately 12.4% of the participants indicated that their family, friends or people around them had recommended that they reduce their consumption at some point. The family may therefore be a potential resource for treatment and rehabilitation [113]. Tobacco use increases this outcome (OR = 3.370). Tobacco has been used as a predictor of other substance abuse, and as such some authors support simultaneous treatment to quit tobacco and other substances [114]. Some studies report that substance use is higher in people with chronic diseases. Wu et al. [115] found that the number of chronic diseases correlated significantly and positively with the severity of drug use. However, in our case, people with chronic illnesses report not using consumption to relax (OR = 0.655). The literature shows patterns of association between substance use, mainly of alcohol, and depressive symptoms [116,117]. A study conducted with adolescents revealed that approximately 5% of the sample openly admitted to using drugs due to sadness, feelings of failure, lack of energy and because they have family problems.

According to our data, stress reduces the probability of getting into trouble for substance use. Low et al. [118] found that stress or health concerns are inversely related to heavy drinking, perhaps due to a reflection that many young adolescents are aware of, and perhaps fearful of, the detrimental health effects of drinking.

Twenty percent of the sample reported consuming alone. In our research this situation increases among people living alone, users who are also smokers and with symptoms of depression. Some authors indicate the importance of ascertaining the context where it takes place in order to understand the risks [119]. A person who consumes alone tends to manifest negative affective traits and depressive symptoms to a greater extent [120]. The predictive model identified in our study has attempted to relate the social and personal consequences of substance use/abuse to some well-known sociodemographic and lifestyle variables, such as age, sex, smoking, stress level and depressive symptoms [94,100,103,104,109,110,114,116,117,118,120], while others are new, e.g., living alone or with family or a friend, the presence of chronic disease, sedentary lifestyle and perceived health status. These warrant further research in order to establish health-promotion strategies and policies for individuals who are more vulnerable or present risk factors to the adverse personal and social consequences of drug abuse. The COVID-19 pandemic began during the study, after which many people changed their lifestyles, and many started to work from home or lost their jobs. These factors may have contributed to the increase in using certain substances. The COVID-19 pandemic has led to measures of social distancing and confinement around the world to reduce the spread of the SARS-CoV-2 virus. These measures could lead to an unpleasant experience after separation from friends and family, and the limitation or prohibition of many daily activities (work, leisure, schools, sport). This situation may induce stress and lead to mental health problems, including an increase in the use of toxic substances [121,122,123]. According to a study conducted through a survey of 3632 participants from Belgium, during confinement, respondents consumed slightly more alcohol and smoked slightly more cigarettes compared to the previous period without confinement [122]. With respect to cannabis, no significant changes in consumption were observed in study performed in Belgium. In Spain, however, data from the Spanish Observatory on Drugs and Addictions (OEDA)-COVID 2020 survey [124], part of the National Plan on Drugs (DGPNSD) of the Ministry of Health and conducted by telephone interviewing 8780 people, show that the measures imposed to control the pandemic have especially reduced the consumption of cannabis (down 17%) and alcoholic beverages (down 8%), especially in young people, in whom the drop in alcohol consumption was as much as 25%. High alcohol consumption in the form of alcohol intoxication also decreased during the pandemic (prevalence of alcohol intoxication before the pandemic was estimated around 17.2% and during the pandemic 9.7%; the decrease in the 15–19 years’ age group was 25%). According to this survey, tobacco consumption decreased in Spain during the pandemic (prevalence of consumption before the pandemic 29.1% and during the pandemic 27.7%), except among women aged 25–34 years and 45–54 years, groups in which prevalence remains more or less stable. With respect to the use of hypnosedatives without prescription, an increase in use was observed during the Covid-19 pandemic (prevalence of use before the pandemic 1.9% and during the pandemic 3.1%). This increase was observed in all age groups and in both sexes, being higher in women. The OEDA-COVID (2020) survey also reveals that cocaine use, which is relatively rare, decreased during the pandemic, from 1.4% before the pandemic to 1% during the pandemic. On the other hand, with respect to persons over 64 years of age who were surveyed, the data show a decrease in the consumption of alcoholic beverages, from a prevalence of 34.2% to 31.8%. With respect to tobacco consumption, there was also a decrease in consumption during the pandemic, from a prevalence of 10.9% to 8.9%. The use of other psychoactive substances such as non-prescription hypno-sedatives, non-prescription opioid analgesics, cannabis and cocaine was not detected in this population group.

## Figures and Tables

**Table 1:** Sociodemographic and lifestyle characteristics of the sample.

Variable	Percentage
Gender	
Man	15.7%
Woman	84.3%
Age (years)	33.61 ± 13.03
Cohabitation	
Alone	7.7%
I share a home with people with no family ties	7.8%
Couple	17.8%
Family	66.7%
Nationality	79.3%
Spain	20.7%
Other	
Do you have a partner?	32.6%
No	67.4%
Yes	
Marital status	51.9%
Single	42%
Married/cohabitation with a partner	5%
Divorced/Separated	1.1%
Widower	
Work activity	2.9%
House master	3.2%
Unemployed	35.2%
Student	1.6%
Retired	0.8%
Worker in the primary sector (agriculture, livestock, fishing and mining)	2.5%
Secondary sector worker (crafts, industry, construction and energy production)	
Tertiary sector worker (commerce, communications, finance, tourism, hospitality, leisure and culture and administration and public services, health and education).	53.8%
Physical activity (*p* < 0.05)	43.8%
No	56.2%
Yes	
Sedentary (*p* = 0.68)	21.1%
No	78.9%
Yes	
BMI	5.9%
Underweight	55.3%
Normal	25.7%
Overweight	13.1%
Obesity	
Chronic Illness	64.3%
No	35.7%
Yes	
Consumption of Stimulant Drinks	23.1%
No	76.9%
Yes	
Tobacco	83.1%
No	16.9%
Yes	

Age is expressed as mean ± standard deviation (SD).

**Table 2:** Descriptive for CRAFFT total score and Substance use (CRAFFT-A) and consequences of its consumption using CRAFFT-B score.

Substance Use (CRAFFT-A)	General	Men	Women
No	43.0%	32.8%	44.9%
Yes(≥1 substance)	57.0%	67.2%	55.1%
CRAFFT_A1. Alcohol			
No	47.9%	37.4%	49.8%
Yes	52.1%	62.6%	50.2%
CRAFFT_A2. Marijuana or hashish			
No	85.3%	83.1%	85.7%
Yes	14.7%	16.9%	14.3%
CRAFFT_A3. Other psychostimulant substances			
No	91.5%	86.8%	92.4%
Yes	8.5%	13.2%	7.6%
	Mean ± SD	Confidence interval for the mean (95%)	
CRAFFT total	2.97 ± 1.78	LL 2.83 UL 3.11	
CRAFFT-B	1.37 ± 1.48	LL 1.27 UL 1.47	

SD: standard deviation; LL: lower limit; UL: upper limit.

**Table 3:** Age (years) differences in terms of consequences of substance use (CRAFFT-B).

Items CRAFFT—B	Mean (±SD)	*p*-Value
B1 No Yes	34.76 (±13.30) 30.21 (±11.50)	*p* = 0.000
B2 No Yes	33.50 (±13.01) 29.27 (±12.08)	*p* = 0.001
B3 No Yes	33.74 (±13.15) 31.73 (±12.44)	*p* = 0.028
B4 No Yes	33.44 (±13.07) 29.13 (±11.35)	*p* = 0.012
B5 No Yes	35.71 (±1 3.16) 27.45 (±10.68)	*p* = 0.000
B6 No Yes	32.96 (±13.08) 33.84 (±12.52)	*p* = 0.181

SD: standard deviation.

**Table 4:** Social consequences according to the substance used.

	Not Used	Alcohol (a)	Marijuana (With or Without Alcohol) (b)	Other Psychostimulant Substances (With or Without Alcohol, and/or Marijuana) (c)
B1. Have you ever traveled in a CAR or vehicle driven by a person (including you) who has consumed alcohol, drugs or psychoactive substances? Yes	* (b) (c) 10%	26.9%	53.1%	52.4%
B2. Have your FRIENDS or your family ever suggested that you reduce your consumption of alcohol, drugs or psychoactive substances? Yes	* (b) (c) 3.9%	11%	23.6%	21.4%
B3. Have you ever used alcoholic beverages, drugs or psychoactive substances to RELAX, to feel better about yourself or to join a group? Yes	* (b) (c) 9.5%	30.3%	44.1%	50.5%
B4. Have you ever gotten into TROUBLE or problems with alcohol, drugs or psychoactive substances? Yes	* (b) (c) 3.1%	6%	11.0%	15.0%
B5. Have you ever FORGOTTEN what you did when you took alcohol, drugs or psychoactive substances? Yes	* (b) 13.7%	32.0%	54.3%	39.4%
B6. Have you ever consumed alcohol, drugs or any psychoactive substance, finding yourself ALONE and without company? Yes	* (b) (c) 3.9%	19.0%	31.5%	47.0%

Data are expressed as percentages.; (*) Differences in non-consumption with categories (b) and (c).

**Table 5:** Mean score on the perceived health status scales, perceived stress scale and anxiety and depression scale according to consumption.

Scale	No Substance Use	Substance Use	*p*-Value
Perceived health statuss	6.760 (±1.698)	7.150 (±1.491)	0.005
Perceived stress scale (EEP-10)	20.563 (±5.123)	20.349 (±5.279)	0.222
Total score in Goldberg scale	12.350 (±3.177)	11.960 (±3.225)	0.089
Score in the Anxiety subscale (Goldberg scale)	7.332 (±1.655)	7.177 (±1.708)	0.232
Score in the Depression subscale (Goldberg scale)	5.020 (±2.107)	4.7779 (±2.153)	0.112

Data are expressed as the mean ± standard deviation for each scale.

**Table 6:** Mean score for anxiety, depression and perceived stress as a function of substance use.

Scale	No Use	Alcohol	Marijuana (With or Without Alcohol)	Other Psychostimulant Substances (With or Without Alcohol, and/or Marijuana)
Score in the Anxiety subscale (Goldberg scale)	6.96 (±1.80)	6.73 (±1.82)	6.74 (±1.75)	7.26 (±1.90)
Score in the Depression subscale (Goldberg scale)	4.81 (±2.13)	4.51 (±2.15)	4.53 (±2.06)	4.87 (±2.20)
Total score in Goldberg scale	12.34 (±3.16)	11.81 (±3.21)	11.67 (±3.24)	12.68 (±3.19)
Perceived stress scale	17.99 (±6.05)	17.30 (±6.02)	17.95 (±6.19)	18.77 (±7.03)

Data are expressed as the mean ± standard deviation for each scale.

**Table 7:** Explanatory variables included in the initial model.

Explanatory Variables	Categories
Age (Years)	Numerical Variable
Gender	1 Man 2 Woman (*)
Cohabitation	1 Alone 2 I share a home with people without family ties 3 Couple 4 Family (*)
Sedentary	No (*) Yes
Chronic illness	No (*) Yes
Tobacco	No (*) Yes
Stimulant drinks	No (*) Yes
Perceived health status	Numerical variable (Range 0–10)
Anxiety subscale (Goldberg)	Numerical variable (Range 0–9)
Depression subscale (Goldberg)	Numerical variable (Range 0–9)
Perceived stress scale	Numerical variable (Range 0–40)

(*) Reference category in regression analysis.

**Table 8:** Final logistic regression model: Variables associated with consumption.

	*p*-Value	Exp(B)	95% C.I. Para EXP(B)	
			LL	UL
Age (years)	0.001	0.975	0.961	0.989
Sex (1)	0.079	1.585	0.947	2.653
Cohabitation: Family	0.068			
Cohabitation (1): Alone	0.708	1.131	0.595	2.147
Cohabitation (2): I share a home with people without family ties	0.016	2.336	1.173	4.652
Cohabitation (3): Couple	0.163	1.360	0.883	2.094
Consumption of stimulant drinks (1)	0.009	1.677	1.138	2.472
Tobacco (1)	0.000	2.579	1.642	4.05
Sedentary (1)	0.058	1.499	0.986	2.279
Health condition	0.005	1.158	1.045	1.283

LL: lower limit; UL: upper limit. Model fit χ^2^ Hosmer-Lemeshow = 2315 *p* = 0.970.

**Table 9:** Final logistic regression model: Variables associated with consequences of consumption.

	B1		B2		B3		B4		B5		B6	
	*p*	Exp(B)	*p*	Exp(B)	*p*	Exp(B)	*p*	Exp(B)	*p*	Exp(B)	*p*	Exp(B)
Age (years)	0.000	0.959	-	-	-	-	0.018	0.948	0.000	0.930	-	-
Gender (1)	-	-	-	-	-	-	0.014	3.031			-	-
Cohabitation: Family	-	-	-	-	-	-	-	-	-	-	0.029	
Cohabitation (1): Alone	-	-	-	-	-	-	-	-	-	-	0.097	2.119
Cohabitation (2): I share a home with people without family ties	-	-	-	-	-	-	-	-	-	-	0.390	0.696
Cohabitation(3): Couple	-	-	-	-	-	-	-	-	-	-	0.022	2.034
Chronic illness (1)	-	-	-	-	0.081	0.655	-	-	-	-	-	-
Consumption of stimulant drinks (1)	-	-	-	-	-	-	-	-	-	-	-	-
Tobacco (1)	0.042	1.739	0.000	3.370	-	-	-	-	-	-	0.012	2.012
Sedentary (1)	-	-	-	-	-	-	-	-	-	-		
Health condition	-	-	-	-	-	-	0.044	0.767	-	-	-	-
Perceived stress scale	0.065	0.964	-	-	-	-	0.045	0.921	-	-	-	-
Depression Subscale (Goldberg)	-	-	0.006	1.211	0.000	1.232	0.091	1.196	0.046	0.898	0.000	1.234
CRAFFT-A: Alcohol (*)	0.000-	-	-	-	0.001		-	-	0.005		0.001	
CRAFFT-A: Marijuana (with or without alcohol)	0.001	2.658	-	-	0.011	2.063	-	-	0.005	2.330	0.049	1.854
CRAFFT-A: Other psychostimulant substances (with or without alcohol and/or marijuana)	0.000	3.405	-	-	0.001	2.784	-	-	0.024	2.022	0.000	2.965

(*) Reference category in regression analysis. Model fit B1 χ^2^ Hosmer-Lemeshow = 4.431 *p* = 0.816; Model fit B2 χ^2^ Hosmer-Lemeshow = 12.384 *p* = 0.089; Model fit B3 χ^2^ Hosmer-Lemeshow = 8.424 *p* = 0.393; Model fit B4 χ^2^ Hosmer-Lemeshow = 5.044 *p* = 0.753; Model fit B5 χ^2^ Hosmer-Lemeshow = 4.162 *p* = 0.842; Model fit B5 χ^2^ Hosmer-Lemeshow = 6.715 *p* = 0.568.

## Data Availability

The data presented in this study are available on request with scientific purpose from the corresponding author.
